# Selected Determinants of Acceleration in the 100m Sprint

**DOI:** 10.1515/hukin-2015-0014

**Published:** 2015-04-07

**Authors:** Krzysztof Maćkała, Marek Fostiak, Kacper Kowalski

**Affiliations:** 1 University School of Physical Education in Wroclaw, Department of Track and Field.; 2 Gdańsk University of Physical Education and Sport, Department of Track and Field.; 3 Inter-provincial Sports Medical Clinic in Wroclaw.

**Keywords:** horizontal jumps, stride characteristics, acceleration phase, muscle strength

## Abstract

The goal of this study was to examine the relationship between kinematics, motor abilities, anthropometric characteristics, and the initial (10 m) and secondary (30 m) acceleration phases of the 100 m sprint among athletes of different sprinting performances. Eleven competitive male sprinters (10.96 s ± 0.36 for 100 with 10.50 s fastest time) and 11 active students (12.20 s ± 0.39 for 100 m with 11.80 s fastest time) volunteered to participate in this study. Sprinting performance (10 m, 30 m, and 100 m from the block start), strength (back squat, back extension), and jumping ability (standing long jump, standing five-jumps, and standing ten-jumps) were tested. An independent t-test for establishing differences between two groups of athletes was used. The Spearman ranking correlation coefficient was computed to verify the association between variables. Additionally, the Ward method of hierarchical cluster analysis was applied. The recorded times of the 10 and 30 m indicated that the strongest correlations were found between a 1-repetition maximum back squat, a standing long jump, standing five jumps, standing ten jumps (r = 0.66, r = 0.72, r = 0.66, and r = 0.72), and speed in the 10 m sprint in competitive athletes. A strong correlation was also found between a 1-repetition maximum back squat and a standing long jump, standing five jumps, and standing ten jumps (r = 0.88, r = 0.87 and r = 0.85), but again only for sprinters. The most important factor for differences in maximum speed development during both the initial and secondary acceleration phase among the two sub-groups was the stride frequency (p<0.01).

## Introduction

The 100 m sprint can be divided into 3 distinct phases: block start with acceleration, maximum speed, and deceleration (Ae et al., 1992; Brüggemann and Glad, 1990; Shen, 2000). The duration and more insightful breakdown of each phase mainly depend on the level of sprint abilities (Mackala, 2007). The acceleration phase may be subdivided into several sub-phases: the initial or starting acceleration (0–12 m), which is mainly characterised by a constant increase of stride length and the main acceleration (12–35 m). When the acceleration phase is of sufficient length and optimum value of running speed, the sprinter is not able to maintain the maximum speed and a long deceleration phase occurs. Top-level sprinters reach their maximum speed between 50 and 70 m (Ae et al., 1992; Brüggemann and Glad, 1990; Gajer et al., 1999) and are able carry on for another 20 m, although very seldom for 30 m. Thus, a third transition sub-phase (35–60 m) takes place only at the elite level. It lasts until the sprinter achieves the level of maximum running speed. In this phase the sprinter reaches peak stride length, stride frequency, and maximum velocity. The deceleration is marked only in the last 10 m section of the 100 m dash (Brüggemann et al., 1999).

At the beginning of the sprint run, the ability to produce a great concentric force/power and to generate high velocity during acceleration is of primary importance (Bissas and Havenetidis, 2008; Mero et al., 1992). Young (1992), Mero and Komi (1994), and Rimmer and Sleivert (2000) suggested that bounding may be considered a specific exercise using the stretch shortening cycle for the development of acceleration. These exercises have similar contact times as sprinting during the initial acceleration phase. Therefore, the greatest transfer of the explosiveness to sprinting can occur. The sprinter also requires strong leg and back extensor muscles. Maximal strength, acquired in the squat and power clean exercises, has been significantly correlated with sprint performance (Wisloff et al., 2004). In turn, Frye (2000) claimed that the technical model of the initial acceleration phase can be achieved by pushing with the drive leg, which requires a forward body-lean from the ground up. The amount (running distance) of body-lean an athlete exhibits is directly proportional to upper body strength.

The relationship between stride length (SL) and stride frequency (SF) for maximizing running speed in different phases of a 100 m sprint performed by athletes of a different sports level (Delecluse et al., 1995; Mann and Sprague, 1980; Ferro et al., 2001; Gejer et al., 1999; Ito et al., 2006; Letzelter, 2006; Mackala, 2007; Salo et al., 2011) and even untrained athletes (Babić et al., 2011; Chatzilazaridis et al., 2012; Coh et al., 1995; Letzelter, 2006) remains poorly investigated. However, based on this knowledge, it is of interest to compare the speed and stride characteristics of each phase expressed relatively to the level of motor abilities, primarily jumping ability, and strength of lower extremities. It is also extremely important to specify the relationships between the kinematic variables of the different phases of acceleration (Coh et al., 2001; Mann et al., 1984), the entire 100 m sprint, body height, body mass, as well as leg length among athletes of different sprinting abilities. Furthermore, this comparison will provide information concerning the efficiency of the acceleration phase.

Most studies provide a general concept for interaction between SL and SF during a 100 m sprint, as the entire course and divided into specific phases in elite and advanced sprinters. Some studies claim that SL is the most significant variable for maximal speed development, whereas others claim that it is SF. The novel aspect of the current study is the analysis of the interaction between SL and SF for maximizing sprinting speed in different phases of a sprinting task performed by athletes of a different sports level. Furthermore, it is of interest for sprint training to compare the stride characteristics expressed relatively to anthropometric variables typical of sprinters such as: body height, body mass or lower limb length among sprinters of a different level of performance. In addition, it is also required to take into account the relationship between different horizontal jumping tests and sprinting performance. This comparison will provide necessary information concerning force production effectiveness for propulsion in order to perform an optimal sprinting step during the acceleration phase or the entire distance of 100 m.

Therefore, the goal of this study was to investigate the relationship between kinematics, motor abilities, anthropometric characteristics, the initial (10 m) and secondary (30 m) acceleration phases of a 100 m sprint among athletes of different sprinting performances. It was hypothesised that different types of horizontal jumps — standing long jump, five jumps, and ten jumps — would correlate differently with speed and stride characteristics (number, length, frequency) across the 10 m initial acceleration, 30 m secondary acceleration, and entire 100 m sprint, regardless of the level of sprint performance. The second hypothesis suggests that the relationship between time and stride characteristics in sprinters with lower sprint ability may represent a bigger dispersal of individualised combinations between SL and SF. Additional information could be also retrieved with the use of cluster analysis. The main reason for the application of this statistical tool was to find which and how significant (large or small) groups of features determined performance of individual sections of acceleration and the entire 100 m sprint between sprinters of a different sports level.

## Material and Methods

### Participants

The study was conducted on a sample of 22 male athletes (aged 21.7 ± 1.08 years; body height 180.8 ± 6.98 cm; body mass 76.6 ± 7.62 kg), who regularly trained or attended athletics classes. The participants were divided into two groups: high performance sprinters (n=11) and a control group of physical education students (n=11). The criteria for student selection were age, active participation in school athletics program, and excellent physical condition at the time of the experiment. The sprinters were involved in regular training for a minimum of five years and specialised in the 100 and 200 m sprint. All subjects were informed on the experimental protocol prior to testing and signed an informed consent form. The study was approved by the Human Ethics Committee of the University School of Physical Education in Wroclaw.

### Experimental Protocol

Testing was conducted on a synthetic track and in a weight room. The sprinter group was tested at the beginning of their competitive season, the physical education students were tested at the end of their school year. The test sessions were conducted in the morning and afternoon with six hours of rest between the 2 sessions. In the morning subjects performed strength and jumping tests. In the afternoon session they performed sprint tests which corresponded to the regular athletes’ sprint training routine. All subjects were familiar with the testing procedures and jumping exercises, which they had already performed as part of their athletic training and athletics classes. The athletes were instructed to be adequately hydrated, fed, and rested prior to and during the testing day. Before each trial, subjects were allowed to perform an adequate standardised warm-up which included light jogging, a stretching routine, light jumping exercises, and accelerations. Regardless of testing modality, the subjects were requested to exert maximal effort in each test. A 2 min rest period was held between jumping trials and a 5 min rest interval between sprints to avoid the effects of fatigue.

**Figure 1:**
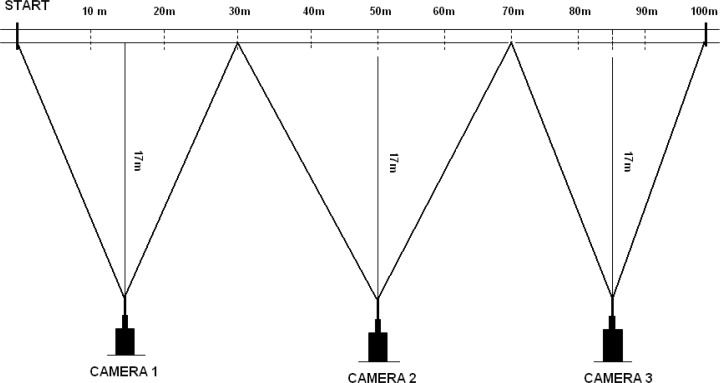
The schematic setup of the speed measurement system

Kinematics of a 10 m, 30 m, and 100 m sprint from the block start were collected using the SIMI Motion System (SIMI Reality Motion Systems GmbH, Germany) from three digital video cameras (JVC GR-DVL9800) set at 25 Hz, than analysed frame-by-frame. The cameras were placed perpendicularly to the running direction at 15 m, 50 m, and 85 m intervals (Figure 1). The cameras were synchronised with the starters gun to measure all necessary kinematic variables. Step length was defined as the period between the toeoff of one leg and ipsilateral toe-off of the next leg. The toe-off was determined from raw data of the last frame when the foot lost contact with the ground. Therefore, stride length was defined as the horizontal displacement of the athlete’s body between consecutive foot take-offs. Stride frequency was calculated as the inverse of time necessary to complete one stride cycle including the flight and stance phases.

### Data collection

#### Anthropometric Measurements

Body mass was measured by means of an electronic scale (SEKA, Axis, Gdansk, Poland) accurate to the nearest 0.5 kg. Measurements were made at the beginning of the experiment, before the warm-up, in training apparel without shoes. For reliability, measurements were repeated twice. Body height and lower limb lengths (from the trochanter of the femoral bone down to the base) were measured by means of an anthropometer (Martin Metal Anthropometer) accurate to 0.5 cm. Additionally, the BMI and stride index were calculated.

#### Trunk Strength Evaluation

Spinal muscles strength was assessed during a loaded trunk flexion measurement using a Spring Dynamometer (DSM - 1000 N, Merzaet SA, Poland) with analog readout and stretching function. It had an error of 2% of reading accuracy and the retention function of the measurement result. The dynamometer was attached by a steel cable (which helps to avoid stretching) to a special hitch in the floor. The subject, in the forward trunk lean position (about 90–95° and almost full extension in the knee joint), held the handlebar of the dynamometer with fully stretched out arms (position similar to the dead lift). The steel cable was taut. The subject slowly straightened his trunk to an upright position, extending the cable of the dynamometer maximally, while holding his arms outstretched. In order to avoid excessive deviation of the trunk from the perpendicular, the subject touched the wall in the final phase of extension. When the subject reached the vertical position and was stabilized, the dynamometer result was recorded. Because of this accommodation, it allowed for maximal loading throughout the entire range of motion. This allowed for the most efficient way to load a muscle. The warm-up consisted of stretching and the performance of two sub-maximal loads. In addition, the ballistic strength (power) of subjects was assessed using a reverse overhead 2 kg medicine ball throw in the supine position. For evaluation of flexibility the sit and reach test was also performed. Athletes performed three consecutive repetitions of the Trunk Flexion Dynamometry test.

#### Maximal Strength (1RM) Evaluation

The 1RM back squat was used to assess maximal lower body strength. A warm-up of 6 to 8 repetitions was performed using 40–60% of the perceived 1RM. After a 1 min rest period, a set of 2 to 3 repetitions was performed at 60–80% of the perceived 1RM. Subsequently, 3 to 4 maximal trials (1-repetition sets) were performed to determine 1RM. Each participant descended to the “parallel” position in which the greater trochanter of the femur was aligned with the knee and ascended until full knee and hip extension. An attempt was considered successful when the movement was completed through a full range of motion without deviating from proper technique and form. Rest intervals between trials were 2 to 3 min. A complete range of motion and proper technique was required for each successful 1RM trial.

#### Jumping Ability Evaluation

The power of the lower extremities of the sprinters was assessed by means of three types of horizontal jumps executed in the following order: a standing long jump (SLJ), standing five-jumps (SFJ), and standing ten-jumps (STJ). From three trials the best result was taken for further analysis. The rest interval between each jump lasted about 1 min and 5 min between the different jumps. From the erect position the subject executed the jump as far as possible (measurement accurate to 1.0 cm), landing on both feet without falling backward using the long jump pit. At the moment of take-off the feet were in the parallel position. Reliability was assessed by intra-class correlation coefficients (ICC) calculated for all 3 consecutive horizontal jumps of each jump type (SLJ, SFJ and STJ). Test-retest reliability coefficients were: R= 0.95 for the standing long jump, R= 0.91 for standing five jumps, and R= 0.92 for standing ten jumps. The high reliability coefficients indicated that the applied test represented consistent horizontal jump performance data for both sprinters and students.

### Statistical Analysis

Standard statistical methods were used for the calculation of means and standard deviations (SD). The relationship between variables was determined using the Pearson product-moment correlation. An independent Student t-test was used to examine the differences in anthropometric characteristics, sprinting speed of the acceleration phase, and explosive power of the lower extremities between the sprinters (n=11) and students (n=11). The intra-class correlation coefficients (ICC) test was used to examine the reliability of horizontal jumps. Additionally, the Ward method of hierarchical cluster analysis based on the linkage distances was applied to determine relatively homogeneous groups of athletes of a different level of motor abilities and anthropometrics characteristics. Statistical significance was set at alpha equal to 0.05, with p ≤ 0.05. Statistical power was determined to be > 0.90 at the 0.05 alpha levels.

## Results

The groups were very similar in age, body height, and the body mass index (BMI). The only exceptions were the variables of body mass and leg length which were on the average of statistical significance (p = 0.0324 and p = 0.0145, respectively). Regarding the kinematic parameters of initial acceleration (10 m), there were no statistically significant differences between the two groups except for the ratio between leg length and stride length (stride index – SI). Contrary to this, the kinematics of 30 m acceleration and 100 m sprint performance revealed statistically significant differences between the groups in time, peak velocity, maximum SF, and the SI, respectively. For the seven motor tests, only two showed no significant differences between the two groups. These were for the over-head 2 kg medicine ball throw and trunk dynamometry, which assessed upper body strength/power during a loaded TF and extension movement.

Significant correlations were shown between the performances of all three horizontal jump tests, including total distance and 10 m initial acceleration, 30 m acceleration, and 100 m for both time and peak speed in sprinters (Table 2). In the student group this relation appeared only for the 30 m sprint (Table 2). There was a similar relationship between the 1RM back squat and 20 m from the flying start. Strength of the trunk showed a relationship only with the SI, both for 10 m, 30 m, and 100 m in sprinter and student groups. The overhead 2 kg medicine ball throw test showed significant correlations only with the time and speed for 10 m initial acceleration.

Strong relationships between anthropometric characteristics and kinematics of 10 m, 30 m acceleration (Table 3) were observed only in competitive sprinters. It concerned correlations between body height, leg length, the stride number and SL for 10 m acceleration and the 100 m sprint. Leg length showed strong relationships with almost all kinematic variables.

Pearson correlation analysis also indicated that the 10 m, 30 m, and 100 m times were strongly correlated with speed, the SI, and max stride length for sprinters. On the other hand, time for each section was related only with speed and SF in both 10 m and 30 m acceleration phases for the group of students. The analysis revealed a strong relationship between time of the 100 m sprint and most kinematic variables of both 10 m and 30 m only for sprinters.

In comparison between the two groups of participants, based on the emerged clusters (the Euclidean distance on level 4), a closer relationship was observed between the measured features among sprinters (fewer numbers of cluster – 2, and a closer bond distances between features) than in the group of students (more clusters – 4, and a larger distance between the binding characteristics and greater randomness in sequence of joining). In this way, in the group of students three clusters emerged, and in the sprinter group there were only two of them. In the group of sprinters, one cluster was substantially created by stride characteristics (stride number across different running sections and indexes resulting from relative SL and leg length). The second cluster was formed by other features but with typical smaller clusters merging in close proximity. With respect to students, the analysed variables formed one cluster which comprised strength and power of both the lower and upper extremities and the shoulder girdle. Other features placed in a fairly random manner were found in the following two clusters.

## Discussion

The influence of biological attributes (body mass, body height or leg lengths) on stride length and frequency cannot constitute a simple explanation for achieving a higher level of sprinting speed and elements that distinguish faster sprinters from the slower ones (students). However according to Uth (2005), Coh et al. (2001), and Coh (2007) the anthropometric characteristics typical of sprinters in part might have an influence on relative muscle strength and step length. Especially in sprinters the presence of long lower extremities is found to be advantageous in achieving top performance (Vucetic et al., 2008). This is not consistent with the understanding that SL cannot only be defined by leg length. Mann and Sprague (1980) along with Simonsen et al. (1985) recognised other factors besides leg length including the force of the extensor muscles of the ankle and knee joints since they produce the push-off impulse in the contact phase.

This experiment revealed that with regard to SL expressed in relation to leg length, the SL ratio was significantly different among groups (r = −3.35, p = 0.0032), but only for 10 m initial acceleration. However, the SL ratio was significantly correlated with time during the 10 m acceleration, 30 m acceleration, and the entire 100 m race for sprinters (r = 0.70, r = 0.83, and r = 0.69, respectively). The SF showed no significant differences between the two groups. Thus, not only the length of the lower limbs, but above all the strength of lower extremities developed with several years of regular training resulted in a stronger impulse during the push-off which automatically induced a longer step among the sprinters. In turn, the lack of a strong correlation between time and stride characteristics in the student group may indicate that athletes with a lower level of sprint ability represent greater individual dispersal of combinations between SL and SF. This caused difficulty in reaching a higher maximal running speed for each section. The longer the running (phase) and the more the race progresses, the greater the number of stride characteristic combinations and the worse the performance.

Research has shown that untrained and low level sprinters reach maximum running speed around 30–40 m (10) and cannot maintain this speed after the 40–50 m mark of a 100 m sprinting test (Coh et al., 2001; Delecluse et al., 1995). Therefore, the secondary acceleration is completed after 20–30 m (Kotzamanidis, 2003; Mackala, 2007). No differences were found between sprinters and students in the 10 m of initial acceleration. This could be attributed to the fact of a sharp increase in running speed due to the lack of abilities to gradually build up speed over the entire acceleration phase in the untrained participants. However, greater differences were observed between groups in the 30 m secondary acceleration times. The fact that the sprinters were able to gradually build up speed suggested that they had a longer acceleration phase, and achieved better performance in a sprinting task (Letzelter, 2006; Mackala, 2007). This could be explained by a significant increase in both SL and SF that allowed sprinters to better coordinate the actions of their body segments in order to accomplish the technique requirements of acceleration. This process had been noted in other studies (Baumann, 1985; van Coppenolle et al., 1985).

Sprinters with higher acceleration ability had a 10.8% and 5.9% higher SF, respectively, for 30 m secondary acceleration and the entire 100 m sprint as compared to the student group. Small differences (2.8%) were observed between groups but concerned only the 10 m initial acceleration. We expected significant differences in SL, especially when the two groups of athletes differed in LL. Some studies have shown that stride frequency specifically influenced the speed of sprint running in track and field (Donatti, 1995; Ferro et al., 2001; Mann et al., 1984; Mero et al., 1992) and in many field sports (Murphy et al., 2003) which may refer only to short sprints up to 40 m. Murphy et al. (2003) confirm by claiming this statement that athletes who are able to generate higher sprint speed over short distances do so because of better stride frequency. This is possible only when a sprinter is able to reduce contact time during the stance phase of a single running stride. The absence of a significant correlation between LL and SL, or SF may indicate the interference of other variables than LL in shaping the kinematic trend of sprinters. Coh et al. (2007) defined SL as a very complex kinematic variable that depends on many factors besides morphological characteristics (leg length), such as muscle structure, reflex mechanisms, and ground force in the propulsion phase, as they are of particular importance in speed development. The most important generator of differences in maximum speed development during both initial and secondary acceleration phases among the two sub-groups was SF (p<0.01). This is quite surprising, and it was not expected that the crucial factor in the difference of quality of the acceleration phase would be a disparity in SF. Thus, the faster sprinters probably are able to achieve higher running speed by striking the ground with greater force and much quicker than slower students do. This is probably another mechanical element that distinguishes fast sprinters from slower ones or untrained students. Weyand et al. (2000) indicate that at top speed, every sprinter takes around a third of a second to pick their foot up and put it down again. It can be concluded that maximum running speed is largely determined by how much force a sprinter can apply to the ground during each step.

An application of vertical and horizontal types of jumping exercises could contribute to longer SL (Coh and Mackala, 2013; Kotzamanidis, 2003; Mackala et al., 2012). However, a strong relationship between these variables has not been established in athletes (Farrar and Thorland, 1987), which is consistent with our research. The results of these experiments have confirmed that a strong correlation exists between maximal strength, horizontal jumping performance, and speed in the group of sprinters in both the 10 m initial acceleration and 30 m secondary acceleration phases. The recorded times of 10 m and 30 m proved that the strongest correlations were found between the standing long jump, standing five jumps, standing ten jumps, and speed in 10 m (r = 0.66, r = 0.72, r = 0.66, and r = 0.72, respectively). It clearly explains why any relationship between these variables in the group of student athletes was not significant. Our experiment also revealed a strong relationship between the 1RM back squat and standing long jump, standing five jumps and standing ten jumps (r = 0.88, r = 0.87 and r = 0.85, respectively), but again only for sprinters. A higher level of maximal strength in the sprinters group automatically results in more powerful horizontals jumps, and 20 m from a flying start. Additionally, information could be retrieved regarding the effectiveness of transforming the skills and level of performance of horizontal jumping ability into acceleration in sprinting. Bounding exercises have been found to possess ground-contact times very similar to those of the acceleration phase of sprinting performance. These results were simultaneously confirmed by two research groups. Wisloff et al. (2004) reported a correlation between 10 m sprint time and 1RM in a free weight squat in soccer players of r = 0.94, and McBride et al. (1999) found a statistically significant correlation between 10 yards sprint time and 1RM/BM (r = 0.54, power = 0.62, p = 0.02) as well as in the 40 yards sprint time and maximal squat 1RM/BM (r = 0.60, power = 0.75, p = 0.01). Similarly, Hunter et al. (2005) and Maulder et al. (2006) also reported a significant correlation between ground reaction force, horizontal impulse, and sprinting velocity (r = 0.78).

The dispersion characteristics of the untrained student group are larger compared with competitive sprinters. However, in both cases the individual clusters formed similar and characteristic agglomerations. It should be emphasised that the features are essentially linked with body height variables (body height and leg length) and stride characteristics (stride numbers, SF, and SL) for different running sections (from 10 m, via 30 m to 100 m). It seems logical that these variables are accompanied by body mass and age, which directly determine the level of human strength capabilities. This distribution and the additional cluster in this group compared with athletes specialising in sprinting may indicate larger disparities and lower speed abilities of the students. We should remember that speed abilities are strongly determined by genetics and that running performance is a rather natural and relatively simple form of movement. The smaller clusters are formed by variables of body composition (height and body mass) along with parameters that specify the mean SL and the maximum SL (30 m and 100 m). Other variables are speeds at different sections, the level of upper body strength (muscles of the back and the shoulder girdle), and further variables that specify the power of the lower limbs. Such a hierarchy of features confirms in a clear way the pattern of division of human motor abilities. As far as sprinting is concerned, it shows the spectrum of physical preparation: strength, power and speed training. According to the cluster analysis, the results reached in 10 m initial acceleration, 30 m secondary acceleration, and the entire sprint performance (100 m) remain in direct relation with LL and consequently with SL and the number of strides taken over a specified distance. Given such a natural form of movement structure that sprint running has, there are large differences between the groups of tested athletes. With regard to sprinters the cluster analysis clearly indicated groups of features that seemed to determine to a large extent the final result in the 100 m sprint.

The study had several limitations what could affect further research. A larger number of subjects with much better sprinting quality would create greater statistical power, however, in these days it is difficult to find a great number of fast sprinters. The experiment did not take into account the measure a single leg (L or R) jumping ability in order to express the differences of strength between limbs. Even a stronger relationship with all kinematics of maximum speed during acceleration could be detected. Further studies should consider the analysis of the rates of speed kinematics changes on a step-to step basis (Nagahara et al., 2014) with the division into smaller sections.

## Conclusions

The different types of horizontal jumps — a standing long jump, standing five-jumps, and standing ten-jumps were correlated differently with time and stride characteristics (stride numbers, length, and frequency) across 10 m initial acceleration, 30 m secondary acceleration, and the entire 100 m sprint, regardless of the level of sprint performance. The correlation appeared strong only in the sprinter group, did not show any differences (was on the same level for all jumps), and concerned only time and speed. This is important in terms of the training process, indicating that the structure of the movement in the horizontal jumps is similar to that of the starting acceleration. There was no relationship between jumping performances and stride characteristics (SL, SF, and stride numbers). The lack of a strong correlation between time and stride characteristics in the group of students may indicate that athletes with a lower level of sprint ability represent a larger dispersal of individualised combinations between SL and SF.

## Figures and Tables

**Figure 2:**
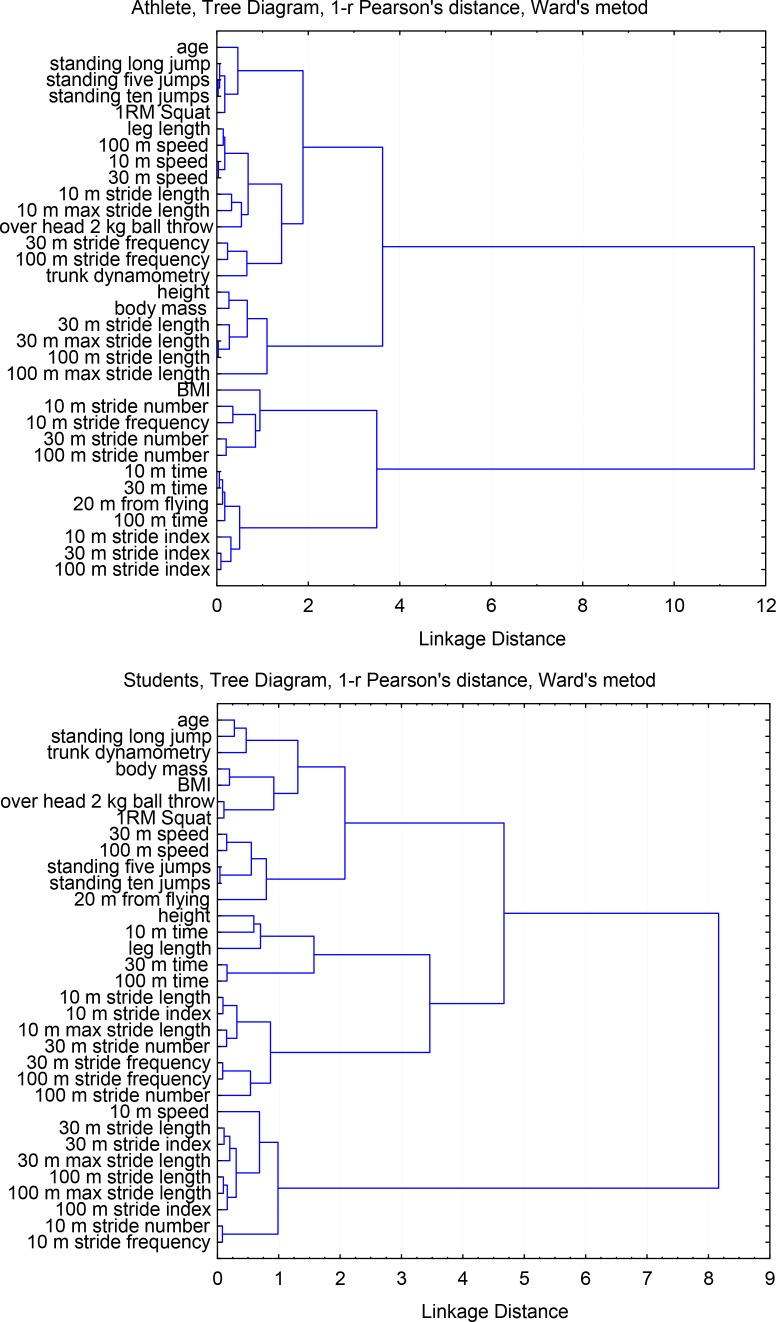
The tree diagram of hierarchical grouping (cluster analysis) considering all anthropometric characteristics and motor abilities in sprinters and students

**Table 1:** Means, standard deviation, and the independent student t-test of anthropometric characteristics, motor ability measurements, and kinematics of 10 m, 30 m acceleration, and 100 m sprint in the groups of sprinters and students (p≤ 0.05)

Variable	Sprinters n=11		Students n=11		t	p
	x̄	SD	x̄	SD		
Antchropometrics						
Age (years)	23.95	2.33	23.18	1.66	0.90	0.3811
Body height (cm)	180.36	6.47	176.36	3.91	1.76	0.0945
Body mass (kg)	75.55	6.01	70.00	5.28	2.30	**0.0324**
Leg length (cm)	90.27	7.35	84.14	1.95	2.68	**0.0145**
BMI	23.16	1.30	22.41	1.34	1.34	0.1960
Kinematics						
10 m time (s)	1.89	0.08	1.93	0.04	−1.70	0.1055
10 m stride number	7.10	0.38	7.10	0.27	0.03	0.9747
10 m stride length (cm)	141.13	7.89	141.73	4.74	−0.22	0.8316
10 m stride index	1.56	0,08	1.67	0.07	−3.35	**0.032**
10 m max stride length (cm)	178.77	7.04	180.11	6.37	−0.47	0.6455
10 m stride frequency (Hz)	3.77	0.19	3.67	0.17	1.21	0.2417
Kinematics						
30 m time (s)	3.93	0.24	4.44	0.11	−6.48	**0.0001**
30 m stride number	16.27	0.65	16.36	0.50	−0.37	0.7170
30 m stride length (cm)	181.09	7.74	177.02	7.05	1.29	0.2116
30 m stride index	2.02	0.13	2.08	0.09	−1.25	0.2271
30 m max stride length (cm)	223.82	9.72	216.73	7.72	1.89	0.0728
30 m stride frequency (Hz)	4.16	0.30	3.71	0.17	4.33	**0.0003**
Kinematics						
100 m time (s)	11.14	0.36	12.20	0.39	−6.68	**0.0000**
100 m stride number	47.64	1.96	48.64	1.91	−1.21	0.2403
100 m stride length (cm)	210.13	8.93	204.05	8.25	1.66	0.1128
100 m stride index	2.34	0.17	2.43	0.09	−1.62	0.1215
100 m max stride length (cm)	239.82	9.03	228.64	11.76	2.50	**0.0212**
100 m stride frequency (Hz)	4.28	0.18	4.03	0.13	3.64	**0.0016**
Physical ability						
Standing long jump (cm)	285.73	15.94	271.36	12.67	2.34	**0.0298**
Standing five jumps (m)	14.65	1.01	13.59	0.60	2.98	**0.0074**
Standing ten jumps (m)	30.68	1.66	28.78	1.75	2.61	**0.0168**
Over head 2 kg medicine ball throw	14.02	2.50	12.51	1.58	1.68	0.1076
Trunk Dynamometry	179.55	27.43	170.91	14.46	0.92	0.3666
1RM Squat (kg)	165.00	34.50	123.64	15.02	3.65	**0.0016**
20 m from the flying start (s)	1.91	0.15	2.15	0.12	−4.20	**0.0004**

**Table 2:** Relationships between (Pearson correlations) the measurements of selected motor tests and kinematics of 10, 30 m acceleration, and the 100 m sprint in sprinters and students

Variable	Sprinters n=11										Students n=11				
	
	[1]	[2]	[3]	[4]	[5]	[6]	[7]	[8]	[9]	[11]	[4]	[5]	[8]	[9]	[11]
Standing long jump	−0.74	0.77	−0.62	−0.70	0.68			−0.82	0.82						
Standing five jumps	−0.65	0.66		−0.62				−0.81	0.80		−0.70	0.73	−0.69	0.73	0.64
Standing ten jumps	−0.71	0.72		−0.67	0.62			−0.83	0.83			0.62		0.62	0.70
Over head 2 kg ball throw	−0.68	0.69													
Trunk Dynamometry			−0.80			−0.67				−0.68					
1RM Squat	−0.71	0.66		−0.65				−0.73	0.73						0.63
20 m run from the flying start	0.89	−0.90	0.76	0.89		0.73	−0.73	0.83	−0.83	0.73					

*10 m time* ***[1]*** *10 m speed* ***[2]*** *10m stride index* ***[3]*** *30 m time* ***[4]*** *30 m speed* ***[5]*** *30m stride index* ***[6]*** *30 m stride frequency* ***[7]*** *100 m time* ***[8]*** *100m speed* ***[9]*** *100 m max stride length* ***[10]*** *100 m stride index* ***[11]***

**Table 3:** Relationships between (Pearson correlations) anthropometric characteristics and kinematics of 10 m, 30 m acceleration in the groups of sprinters and students

Variable	Sprinters n=11														Students n=11
	
	[1]	[2]	[3]	[4]	[5]	[6]	[7]	[8]	[9]	[10]	[11]	[12	[13]	[14]	[14]
Age															0.65
Body height			−0.66	0.67		0.60	−0.62			−0.72	0.72		0.71		
Body mass															
Leg length	−0.75	0.84	−0.76	0.78	−0.65	0.71		−0.87	0.88			−0.76		0.62	
BMI															

*10 m time* ***[1]*** *10 m speed* ***[2]*** *10 m stride number* ***[3]*** *10 m stride length* ***[4]*** *10m stride index* ***[5]*** *10 m max stride length* ***[6]*** *10 m stride frequency* ***[7]*** *30 m time* ***[8]*** *30 m speed* ***[9]*** *30 m stride number* ***[10]*** *30 m stride length* ***[11]*** *30m stride index* ***[12]*** *30 m max stride length* ***[13]*** *30 m stride frequency* ***[14]***

**Table 4:** Relationships between (Pearson correlations) kinematics of 10 m, 30 m acceleration, and the 100 m sprint in the groups of sprinters and students

Variable	Sprinters n=11										Students n=11					
	
	[1]	[2]	[3]	[4]	[6]	[7]	[8]	[9]	[10]	[12]	[2]	[5]	[6]	[7]	[9]	[11]
10 m time (s)	−	−0.96	0.70								−1.00	−0.61				
30 m time (s)	0.94	−0.96	0.77	−0.61		−0.97	0.83	−0.82						−0.95	−0.71	
100 m time (s)	0.83	−0.86	0.71	−0.61	0.90	−0.88	0.63	−0.67	−1.00	0.69						−0.98

*10 m time* ***[1]*** *10 m speed* ***[2]*** *10m stride index* ***[3]*** *10 m max stride length* ***[4]*** *10 m stride frequency* ***[5]*** *30 m time* ***[6]*** *30 m speed* ***[7]*** *30m stride index* ***[8]*** *30 m stride frequency* ***[9]*** *100 m time* ***[10]*** *100 m speed* ***[11]*** *100 m stride index* ***[12]***
